# Exploring the role of green government publicity influencing people’s pro-environmental behaviors

**DOI:** 10.3389/fpsyg.2022.973160

**Published:** 2022-10-20

**Authors:** Yi Lin, Jiechun Li, Ling Xiang

**Affiliations:** ^1^School of Public Administration, Jilin University, Changchun, Jilin, Zhuhai, China; ^2^School of Marxism, Zhuhai City Polytechnic, Guangdong, China; ^3^School of Shipping Economics and Trade, Guangzhou Maritime University, Guangzhou, China

**Keywords:** environmental policy, green packaging, green government publicity, environmental concerns, moral obligation, pro-environmental behaviors

## Abstract

In recent years government publicity was extensively used to convey environmental issues; therefore, it is important to explore the role of green government publicity influencing people’s pro-environmental behaviors (PEBs). This study is to uncover the impact of China’s green government publicity on people’s willingness to use green packaging. This research collected data from Guangzhou of China, we used convenient sampling and online questionnaire survey to gather data, and there were 584 effective samples. Using the statistical software Amos17.0, the results reveal that green government publicity has a significant effect on environmental concerns and moral obligation. Environmental concerns and moral obligation both have positive effects on PEBs. In addition, Environmental concerns and moral obligation all have mediating effects in the relationship between green government publicity and people’s PEBs.

## Introduction

Due to large amount of environmental pollution which directly connected with industrial production in the world, society has found that the issues of environmental protection become more and more important (Weinberger et al., 2015). For example, Ionescu (2021a) found that corporate environmental performance helps people to perform green innovation behavior in sustainable finance. May et al. (2021) integrate the environmental sustainability literature from the perspective of CSR initiative and employees’ green behavior. Lăzăroiu et al. (2020) confirm the relationship between sustainable development governance, organizational knowledge, sustainable organizational development, and corporate sustainability, which influences corporate environmental and sustainability management. Ionescu (2021b) analyze the relationship between green financial behavior, climate change mitigation, and environmental energy sustainability. Dabija et al. (2018) found that Romanian retailers care about attracting customers and gaining their loyalty by adopting strategies based on the principles of sustainability.

In 1980, concept of packaging recycling and reusing emerged, at that time pioneers of European packaging design advocated “greenism” and emphasized the important role of green packaging. Therefore, green packaging became consensus of 1990s. Owing to the enhancement in packaging technique and common people’s revenue, commodity packaging was more and more delicate and complicated. Green packaging stressed the issues of environment, simultaneously pursued environment protection and marketing benefits, and aims at satisfying consumers’ needs and improving environment quality (Weinberger et al., 2015).

With the growing role of environmental protection, capturing drivers of behavior helps us understand more about human behavior which may benefit environmental protection (Steg and Vlek, 2009). In this context, pro-environmental behavior (PEB) is an individual’s behavior to engage in environmental activities to protect the earth (Kollmuss and Agyeman, 2002). Although PEB is positively related to socio-demographic (age, education, income, gender, and environmental knowledge) (Hunter et al., 2004; Johnson et al., 2004; McCluskey et al., 2009; De Silva and Pownall, 2014; Pothitou et al., 2016) and psychological determinants (belief, awareness, value, norms, and emotion) (Zhang et al., 2014; Arimura et al., 2016; Hornsey et al., 2016; Steg, 2016; Ibanez et al., 2017), reviewing related research indicates that government publicity about them is still limited, posing a research gap in this area.

In recent years, China began to respond to the increasingly prominent ecological and environmental problems. From the perspective of the relationship between environmental issues and environmental policy, government cares about whether or not it can actually put the related policy into practice. In China government publicity is developed to deliver the government’s philosophy. Because the leading role is government, it has more resources such as financial support, a clear schedule, and specific leadership. In recent years government publicity was extensively used to convey environmental issues; therefore, it is important to uncover the impact of China’s green government publicity on people’s intentions to use green packaging. The research question is to uncover the role of China’s green government publicity influencing people’s attitudes and behaviors to comply with the environmental protection regulations. The aim is to explore people’s attitudes and behaviors to participate in green packaging activities based on the crucial role of green government publicity in China.

To fill the research gap, this study use the theory of stimulus–organism–response (SOR) to explore the role of green government publicity influencing people’s PEBs. We applied SOR to examine the stimuli of green government publicity to promote PEBs *via* the mediating roles of environmental concerns and moral obligation. This research uses SOR to propose that green government publicity (S) will cause an internal evaluation of someone (O) (environmental concerns and moral obligation) and then produce a response (R) (PEBs). This research established a framework and suggested that green government publicity plays an important role determining environmental concerns and moral obligation, which enhances PEBs.

We contribute to the existing research in some ways. First, few studies explored government publicity in green context, this research used the SOR theory to analyze the impact of green government publicity on environmental concerns and moral obligation, which enhances PEBs to use green packaging. Second, the findings will enable us to have clearer understandings of people’s attitude toward using green packaging and to help the authority-concerned improve the effectiveness of green packaging policy implementation.

The following section of this paper presents a review of the research literature and the research hypotheses. This is followed by the research methodology and measures, results of the study, and a discussion of the findings. The paper concludes with theoretical and practical implications, limitations, and suggestions for future research.

## Literature review and hypotheses development

### Green packaging policy in China

In order to reduce environmental pollution, the China’s government regulates related laws and policies, e.g., they upgrade the express green packaging standard system and support the formulation of mandatory national standards for technical requirements related to the environmental protection. In addition, they accelerate the development of green standards for express packaging, focus on the combination of packaging design and information technology, and promote the incorporation of environmental sensing and traceability technologies into existing express packaging product standards (Yang and Zhao, 2019). According to the amended Solid Waste Law in December 2020, producers must comply with mandatory standards for product packaging. The standards give three technical requirements of packaging specification, such as: the limited amount of package layers, empty space ratio, and the packaging cost percentage of the total price for different product categories. If the packaging does not satisfy any one of the requirements, it will be determined as excessive packaging and will be subject to a fine.

### Green government publicity

When government aims to promote a new policy, it needs citizens to follow the regulation. If citizens do not understand the related rules, it’s difficult for the government to implement the policy. Hence, before implementation, the government should carry out vigorous publicity to help citizens gain related knowledge on the policy, so they can easily to implement the policy (Zhou et al., 2019). Promotional information about green packaging can be seen everywhere in newspapers, television, radio, and on the internet. Each community distributes publicity materials on green packaging to its citizens. In addition, the government takes measures to widely publicize the policy, these measures include incentives and penalties in legal documents (Tian et al., 2019). Extending the concept of government publicity in green setting, green government publicity refers to government’s publicity to help citizens gain environmental knowledge on the policy, so it helps the government easily implement the policy.

### Environmental concern

Environmental concern means the extent of people’s concerns for the evaluation of environmental problems (Schultz, 2001). Environmental concern denotes an individual’s general orientation toward the environment and an individual’s concern level as to environmental issues has been found to be a useful predictor of environmentally conscious behavior (Binder and Blankenberg, 2016). Schultz (2001) proposed that a person’s environmental concerns are based on the degree to his perceived interconnection between himself and other people or between himself and nature. When people concern about the Earth’s ecology rather than individuals’ interests, they are likely to perform behaviors beneficial to environmental protection (Schultz and Wesley, 2000). Environmental concern is an important factor in an individual’s decision whether to conduct green behaviors. An enterprise should master the characteristics of consumers with a higher environmental concern before setting marketing strategies to achieve better effects (Binder and Blankenberg, 2016).

### Moral obligation

Moral obligation refers to the degree to which an individual acts (or not) morally (or immorally) when facing an ethical situation (Leonard et al., 2004). In addition to some variables such as attitude, subjective norm, and perceived behavioral control, some studies regard moral norm as additional independent predictor of intention in the environmental research area (Kai et al., 2018). Prior research suggested that a person’s moral obligation is a determinant of environmentally behavioral intentions (Leonard et al., 2004). The TPB model has been extensively used in a wide variety of behavior; nevertheless, the theoretical model has been criticized for neglecting moral consideration (e.g., Manstead, 2000). A person’s belief about exhibiting a specific behavior is often associated with moral norms. His belief in moral rectitude is related to moral norms while performing a specific behavior (Manstead, 2000). This suggests that an individual’s feeling of moral obligation to perform a certain behavior should be considered (Leonard et al., 2004). Moral obligation has significantly improved the prediction of intentions to act in a moral manner (Leonard et al., 2004).

As public cares more about environmental issues, and increasing social attention and media covering environment issues, people start to pay attention to the environmental sustainability (Kollmuss and Agyeman, 2002). PEBs can be defined that people make efforts to perform some behaviors to protect the environment, such as using nontoxic substances, reducing waste production, and participating in a pro-environmental organization (Snelgar, 2006).

### Research hypothesis

Publicity helps draw the attention of people toward the public activities (Deephouse and Carter, 2005). The effect of government publicity outperforms non-profit organizations (Hirvonen and Headey, 2018), because the influence of publicity is stronger and wider, it may generate people’s concerns for the green issue (Spotts et al., 2015). In addition, promotional information can be observed in television, internet and so on. Each community distributes green packaging publicity materials to citizens, which may draw citizens’ environmental concerns (Spotts et al., 2015). Hence, the hypothesis1 is proposed.

*Hypothesis 1*: Government publicity is positively affecting environmental concerns of using green packaging.

To conduct effective implementation of the policy, the government should publicize green packaging regulations to help people gain relevant knowledge (Hirvonen and Headey, 2018). The characteristics of China’ culture is that the publicity by the government plays an important role in promoting the implementation of related environmental regulations by citizens, and people are willing to voluntarily follow the rules (Meng et al., 2019). In China, the related regulations of green packaging is very extensive in the daily lives (Spotts et al., 2015). People are well educated to learn to use green packaging, and they are asked to this publicity helps build social norms in the society. Under the social norms, the publicity of green packaging is very extensive and all people are affected (Spotts et al., 2015). People are aware of the moral obligation to abide by the related regulations. Therefore, the hypothesis 2 is proposed:

*Hypothesis 2*: Government publicity is positively affecting moral obligation of using green packaging.

None and Datta (2011) proposed that environmental concerns have positive effects on green behavior in India. Environmental concerns are likely to influence people’s environmentally-friendly behaviors (Wheeler et al., 2013). Environmental concern relates positively to PEB (Schmitt et al., 2018), those who concern about the environmental situation tend to perform environmentally-friendly behaviors (Binder and Blankenberg, 2016), such as energy consumption (Urban and Scasny, 2012), sustainable food consumption (Panzone et al., 2016) and so on. When people concerned about values of the Earth’s ecology, they tend to engage in environmental protection behaviors (Snelgar, 2006). Hence, we propose the following hypothesis:

*Hypothesis 3*: Environmental concerns are positively affecting PEBs of using green packaging.

Moral obligation can be defined as the people’s obligation to perform or not perform specific behaviors (Ajzen, 1991). Prior study regards moral obligation as important determinant of intention influencing PEB (Zhang et al., 2015). Those who with moral obligation tend to perform PEBs (Shaw et al., 2016). Therefore, Moral obligation toward green packaging will affect PEBs.

*Hypothesis 4*: Moral obligation is positively affecting PEBs of using green packaging.

Theory of Stimulus-Organism-Response proposed stimulus of the environment will influence people’s psychological stability, thereby promoting changes in their behavior (Fu et al., 2021). This theory suggests that people’s organisms are important factors responding to the environmental stimulus (Deng and Yang, 2022). Based on the model, we suggest that green government publicity is a stimulus (S), it will cause an internal evaluation of someone (O) and then produces a response (R) (PEBs to use green packaging). Environmental concerns and moral obligation are the variables related to internal evaluation, which belong to the nature of attitude, and these two variables are found to influence PEBs (Binder and Blankenberg, 2016; Shaw et al., 2016). Therefore, in this study green government publicity serves as an independent variable, environmental concerns and moral obligation are viewed as mediating variables, and PEBs is regarded as the outcome of internal evaluation. Based on the statement, this research proposes the following hypothesis:

*Hypothesis 5*: Environmental concerns will have mediating roles in the relationship between green government publicity and PEBs to use green packaging.

*Hypothesis 6*: Moral obligation will have a mediating role in the relationship between green government publicity and PEBs to use green packaging.

**Figure 1:**
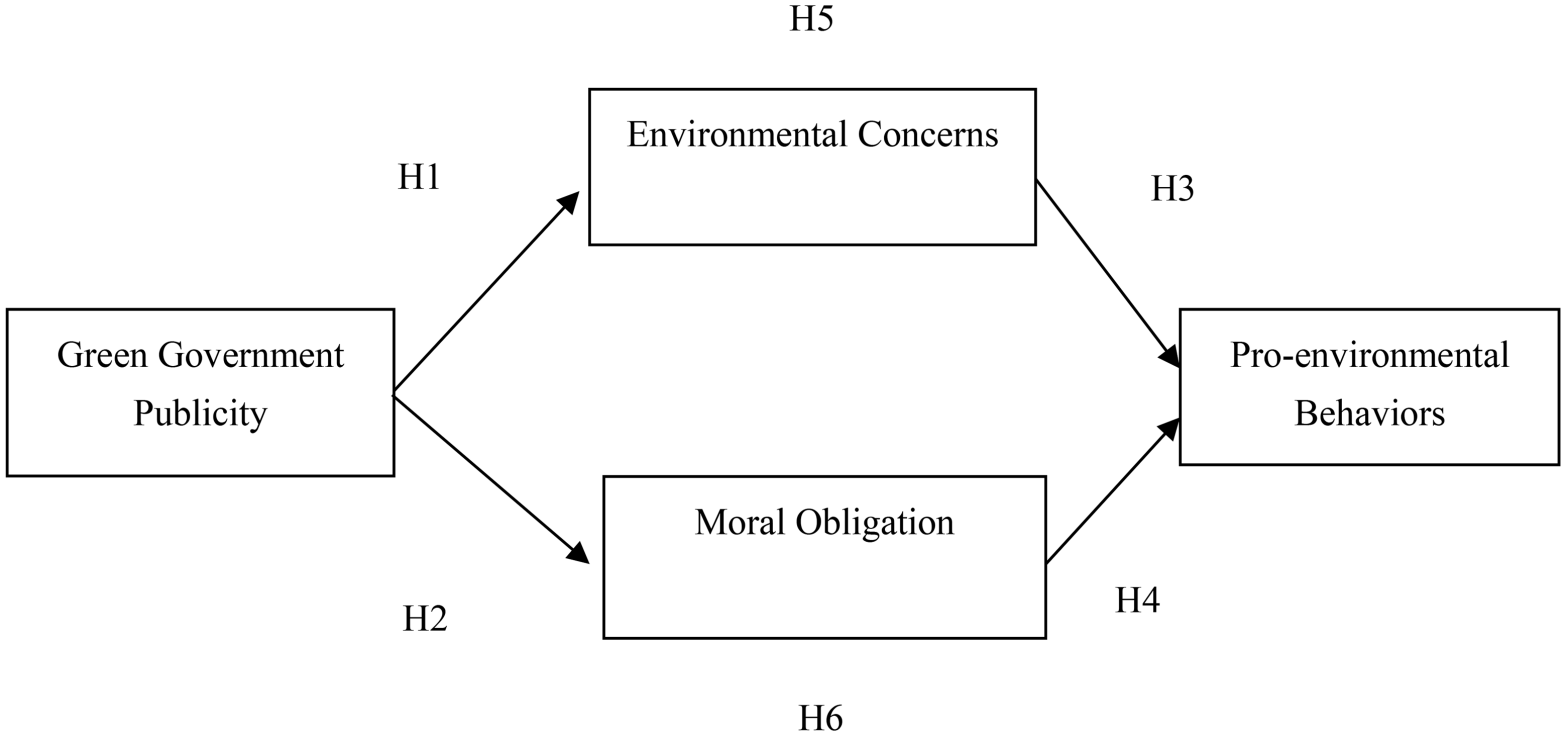
Conceptual model.

Based on the related hypotheses development, we propose the conceptual model (Figure 1).

## Methodology

In Methodology, this study use descriptive statistics to capture the profiles of samples. Then we conduct reliability and validity analyses to check the appropriateness of the constructs. Finally, we use LISREL VIII and bootstrapping to test the hypotheses. The research context is in China, because green packaging regulation has just begun to be implemented on a large scale in China. The government has launched a wide range of publicity strategies, but the effectiveness of the publicity is unknown. Understanding how to enhance the effectiveness of policies can be a benchmarking because it is relatively lacking in the existing research (Meng et al., 2019).

### Sample and procedures

In advance of the formal survey, online questionnaires were sent to 50 people in Guangzhou of China. The 50 people were asked to confirm the meaning of the items and to identify any ambiguities. Then this study gathered data from Guangzhou of China, we used convenient sampling and online questionnaire survey to gather data. The targets are Guangzhou’s people having the experience of using green packaging in China. Finally there were 584 effective samples, suggesting a response rate of 83.4%.

Among the 584 respondents, 62.6% were men and 37.4% were women. Regarding age, 52% of the respondents were aged between 21 and 30 years. Education levels were fairly high, with over 76% having been educated at college level. Most of the respondents (64%) was not married. 44% monthly incomes are between USD $600 to USD $750 (average income in China is about USD $450).

### Measures

Four scales in this study were derived from previous research. Respondents answered the items using a five-point Likert-type scale (1 = “strongly disagree”; 5 = “strongly agree”). The four constructs in the questionnaire included green government publicity, environmental concerns and moral obligation, and PEBs to use green packaging. Green government publicity’s items were from previous study and adjusted to green context (Gao et al., 2017). Environmental concerns were measured by three items (Schultz, 2001). Moral obligation was measured using two items based on previous study (Leonard et al., 2004). PEBs were measured by three items used in previous literature, which were adjusted to suit the context of this research (Zhou et al., 2019).

## Results

### Reliability and validity

Reliability was confirmed by Cronbach’s alphas and composite construct reliability (CCR), all Cronbach’s alphas exceed the suggested value of 0.70 (Hair et al., 2006). Moreover, all the CCR values exceeded 0.7 (Table 1), suggesting suitable composite reliability (Hair et al., 2016). With regard to validity, the study applied convergence and discriminate validity. The mean variance extraction (AVE) for each construct is higher than 0.5 (Schwab, 2006; Table 1), indicating that the scale has convergence validity. In addition, this study measured discriminate validity by calculating the AVE of all structural pairs. The square root of the AVE for each construct was higher than its correlations with all other constructs (Schwab, 2006), which supports discriminant validity (Table 2). Overall, all the constructs in the study performed suitable reliability and validity.

**Table 1:** Measurement model assessment.

Variables and item	Standardized loading	CR	AVE
Green government publicity (GGP) (α =0.861)		0.843	0.64
I see the government publicity of green packaging regulation every day	0.851		
I see the publicity of green packaging regulation in public places	0.852		
I think the government has done a lot to publicize the green packaging regulation	0.849		
Environmental concerns (EC) (α =0.894)		0.823	0.61
I am extremely worried about the state of the world’s environment and what it will mean for the future	0.906		
Humans must live in harmony with nature in order to survive	0.817		
When humans interfere with nature it often produces disastrous consequences	0.803		
Moral obligation (MB) (α =0.931)		0.910	0.63
I have a moral obligation to use green packaging	0.847		
I have an obligation to future generations to use green packaging	0.895		
Pro-environmental behaviors (PEB) (α =0.926)		0.908	0.59
I use green packaging in daily lives	0.907		
I care about green packaging issues	0.892		
I use green packaging to protect the environment	0.918		

**Table 2:** Descriptive statistics and correlations among indicator variables.

Variables	*M*	*SD*	(1)	(2)	(3)	(4)
Green government publicity (1)	4.14	0.75	**0.776**			
Environmental concerns (2)	4.10	0.84	0.456*	**0.814**		
Moral obligation (3)	4.09	0.97	0.378*	0.416*	**0.816**	
Pro-environmental behaviors (4)	4.04	0.92	0.412*	0.312*	0.463*	**0.802**

*n* = 584. Figures along the diagonal (in bold) are the values for the square root of the AVE.

* *p* < 0.05.

**Figure 2:**
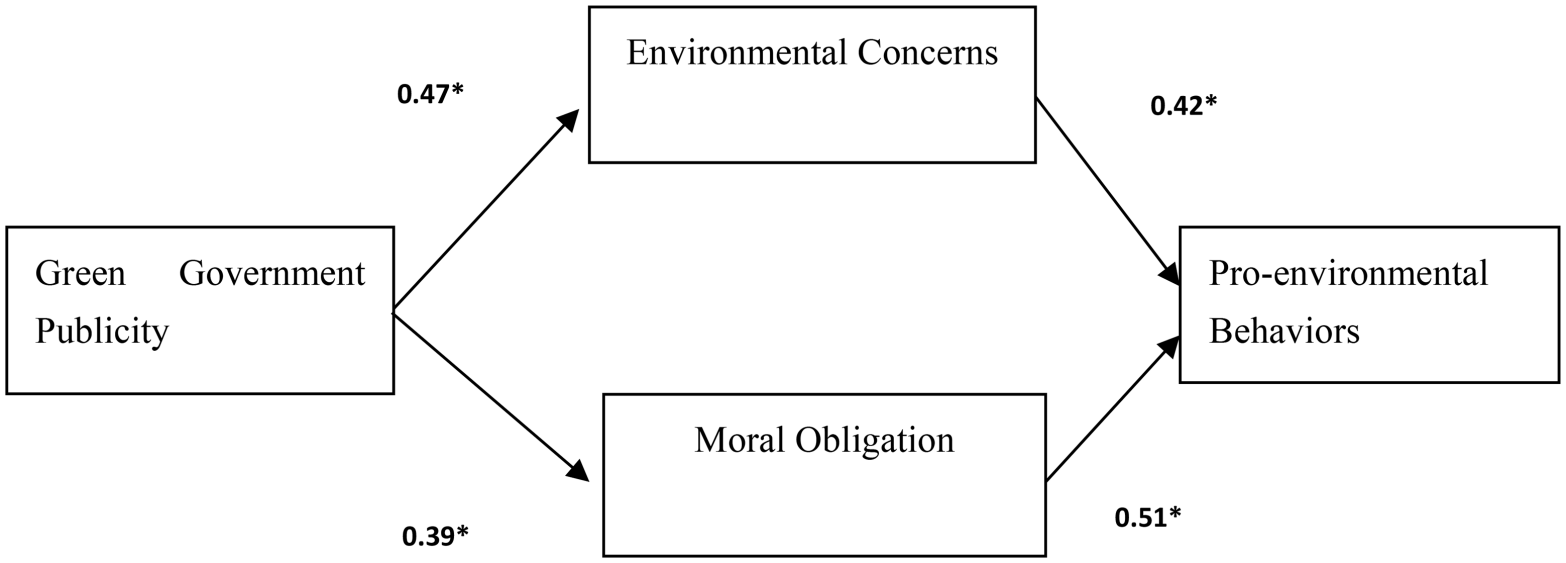
Result of the research model. *p < 0.05.

This research tested the measurement model using LISREL VIII (Joreskog and Sorbom, 1996). To choose the best model, this study uses model competition to compare each model and then chooses the best one according to model fitness. The results indicate that SEM 1 with full mediation effect (Figure 2) (chi-square value/ d.f. = 2.14, CFI =0.93; IFI = 0.93; NFI = 0.92) fit the data better than SEM 2 with partial mediation effect (chi-square value/d.f. = 2.98, CFI =0.89; IFI = 0.87; NFI = 0.87). This indicates that the full mediating model is the best model in this study. All of the indicators are within the acceptable limit, proving that the overall structural model furnished a good fit with the data.

### Hypothesis testing

H1 and H2 hypothesized that green government publicity are positively associated with environmental concerns and moral obligation. As seen in Table 3, the relationships with environmental concerns and moral obligation from green government publicity are fully supported. Hence, the finding suggested that H1 and H2 were fully supported.

**Table 3:** Results of hypotheses and model statistics.

Result	Path coefficient	*t*-value	Results
Green government publicity 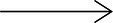 Environmental concerns	0.47	2.20*	Supported
Green government publicity 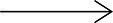 Moral obligation	0.39	2.12*	Supported
Environmental concerns 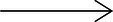 Pro-environmental behaviors	0.42	3.93*	Supported
Moral obligation 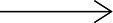 Pro-environmental behaviors	0.51	4.05*	Supported

* *p* < 0.05.

H3 and H4 suggested the impacts of environmental concerns and moral obligation on PEBs. The results proved that environmental concerns and moral obligation have positive influences on PEBs. This indicated that environmental concerns and moral obligation are crucial predictors of PEBs to use green packaging.

Next, we tested mediation hypotheses. Table 4 summarizes bootstrapping results. Environment concerns and moral obligation were found to mediate the relationships between green government publicity and PEBs, as the 95% CIs did not contain zero. Thus, Hypotheses 5 and 6 were supported.

**Table 4:** Results of mediating effect.

Predictor	Mediator	Outcome	95% CI
Green government publicity	Environment concerns	Pro-environmental Behaviors	[0.04, 0.25]
Green government publicity	Moral obligation	Pro-environmental Behaviors	[0.05, 0.27]

## Discussion and conclusion

### Discussion

Due to the growing issue of green packaging, China incorporated related regulations into law enforcement documents. To our best knowledge, few studies explored how to promote people’s behaviors to use green packaging. Therefore, this research uses the SOR theory to analyze the effect of green government publicity on environmental concerns, moral obligation and PEBs to use green packaging. Overall, the results fully confirmed the proposed hypotheses that green government publicity is viewed as a crucial determinant influencing people’s environmental concerns and moral obligation. This research provides an empirical study of the impacts of green government publicity on people’s environmental concerns and moral obligation. Government publicity enables citizens to arouse concerns and moral obligation about green packaging issues, which makes behaviors for green packaging operable. This conclusion shows that government’s publicity related to green issues is relatively effective, with positive effects on people’s concern for the evaluation environmental problems and people’s responsibility to act morally when facing an ethical situation.

Pro-environmental behaviors to use green packaging are determined by environmental concerns. The findings are consistent with the conclusions in other recent literature (None and Datta, 2011; Shaw et al., 2016). Environmental concerns have positive effects on PEBs, which is similar to the results of prior research (None and Datta, 2011). When people are more concerned with the Earth’s ecology, the more likely they are to perform environmental protection behaviors. In addition, moral obligation has a positive influence on PEBs, suggesting that collective consciousness emphasized green packaging in daily life, where individual’s environmentally-friendly behavior tend to be influenced by the moral obligation. The empirical finding is consistent with the results of previous study that moral obligation is an important role in predicting one’s intention to exhibit PEBs (Brody et al., 2012). Si et al. (2020) also found that moral obligation is an important determinant driving environmentally-friendly behavior in China. When an individual has a strong moral obligation, he/she is significantly more likely to perform the behaviors to follow green packaging regulations. The finding of the research did provide suggestions for the government to publicize the related law effectively.

Finally, this study confirmed the mediating effects of environmental concerns and moral obligation in the relationship between green government publicity and PEBs to use green packaging. We used Stimulus-Organism-Response to confirm that green government publicity is a stimulus (S), it will cause an internal evaluation (O) (environmental concerns and moral obligation) and then produces a response (R) (PEBs to use green packaging). The result indicated that green government publicity can lead to more environmental concerns and moral obligations, thereby facilitating people’s PEBs to use green packaging. Therefore, the government can make efforts to use public communication tool to make people in China understand that green packaging is a critical social risk and urgently requires people to take immediate actions. Governments must continue to promote ecological activities through publicity to reform people’s practices. The finding helps us understand people’s psychology about joining in green packaging regulation.

### Theoretical implications

This article has made three theoretical contributions. First of all, although numerous research explored factors influencing PEB (Hunter et al., 2004; Johnson et al., 2004; McCluskey et al., 2009; De Silva and Pownall, 2014; Pothitou et al., 2016); to our best knowledge, few research uncovers the role of government publicity influencing PEB, suggesting a research gap in this area. To fill the gap, government publicity employed in the context of environmental policy was proven to be a determinant of people’s attitudes and environmental behaviors in China. Second, this study extended the concept of government publicity in green setting, this new notion helps us understand more about the tool of green government publicity influencing people’s environmental behaviors. Third, this article considers green government publicity as a crucial factor in the implementation of green packaging. This research constructs a comprehensive framework for exploring the role of green government publicity influencing people’s environmental concerns, moral obligation and PEBs. The findings suggest that green government publicity is the antecedent variable that affects people’s environmental concerns, moral obligation, and PEBs. This study investigates the effect of government publicity of green packaging to help the government allocate resources more effectively.

### Managerial contributions

This research has insights for management. Owing to the growing role of green packaging, how to conduct packaging issues effectively is an important aspect. This research proposes methods and pathways toward the sustainable implementation of green packaging, and the result provides the government with recommendations for effectively managing the implementation of green packaging. Green government publicity is a crucial determinant driving people’s attitudes and behaviors in green activities. The implementation of green packaging policy needs to be vigorously publicized by the government.

### Limitations

The research limitations are described as follows: First, the sample of this study comes from China, so the generalization may be limited. Second, causality could not be determined, future research, preferably using a longitudinal design to provide evidence of the causal linkages proposed in the model. Finally, it is possible that the proposed model may not capture all of constructs that influence people’s PEBs, future studies may work on potential factors that may influence the behaviors.

### Future research perspectives

The study provides some directions for future research. First, future research can take a random approach to select organizations, or across different industries to increase the generalization of the finding. Second, Schwab (2006) believes that researchers can use long-term data to verify the causal relationship of variables, future research can collect long-term data to confirm the relationship. Third, the study used the SOR model to capture the impact of green government publicity on people’s behaviors to use green packaging. Future research adopt more variables, such as personality trait to enrich the research area.

## Data availability statement

The original contributions presented in the study are included in the article/supplementary material, further inquiries can be directed to the corresponding author.

## Ethics statement

Ethical review and approval was not required for the study on human participants in accordance with the local legislation and institutional requirements. Written informed consent from the [patients/ participants OR patients/participants legal guardian/next of kin] was not required to participate in this study in accordance with the national legislation and the institutional requirements.

## Author contributions

YL: conceptualization, methodology, and writing – original draft. JL: software, formal analysis, and investigation. LX: conceptualization, investigation, resources, supervision, writing – review, and editing. All authors contributed to the article and approved the submitted version.

## Conflict of interest

The authors declare that the research was conducted in the absence of any commercial or financial relationships that could be construed as a potential conflict of interest.

## Publisher’s note

All claims expressed in this article are solely those of the authors and do not necessarily represent those of their affiliated organizations, or those of the publisher, the editors and the reviewers. Any product that may be evaluated in this article, or claim that may be made by its manufacturer, is not guaranteed or endorsed by the publisher.
